# Data Resource Profile: Medicines in Acute and Chronic Care in Scotland (MACCS)

**DOI:** 10.1093/ije/dyag187

**Published:** 2026-09-17

**Authors:** Cosmika Goswami, Tanja Mueller, Amanj Kurdi, Ewan Pearson, Khaled Bedir, Alexandra Tolfrey, Hazel Close, Marion Bennie

**Affiliations:** Strathclyde Institute of Pharmacy and Biomedical Sciences, University of Strathclyde, Glasgow, United Kingdom; Strathclyde Institute of Pharmacy and Biomedical Sciences, University of Strathclyde, Glasgow, United Kingdom; Public Health Scotland, Edinburgh, United Kingdom; Strathclyde Institute of Pharmacy and Biomedical Sciences, University of Strathclyde, Glasgow, United Kingdom; Public Health Scotland, Edinburgh, United Kingdom; College of Pharmacy, Al-Kitab University, Kirkuk, Iraq; Department of Public Health Pharmacy and Management, School of Pharmacy, Sefako Makgatho Health Sciences University, Pretoria, South Africa; Faculty of Health, University of Dundee, Dundee, United Kingdom; Faculty of Health, University of Dundee, Dundee, United Kingdom; Public Health Scotland, Edinburgh, United Kingdom; Public Health Scotland, Edinburgh, United Kingdom; Strathclyde Institute of Pharmacy and Biomedical Sciences, University of Strathclyde, Glasgow, United Kingdom; Public Health Scotland, Edinburgh, United Kingdom

**Keywords:** Medicines, Acute Care, Chronic Care, Data Resource

Key Features

- The Medicines in Acute and Chronic Care in Scotland (MACCS) data resource was established in 2025 to integrate medicine-related data with other electronic data from Scottish healthcare systems, creating a national, linked, routinely updated data resource at the population level.
- MACCS provides pre-linked data from multiple routinely collected national datasets within NHS Scotland, including, but not limited to, prescribing records, hospital episodes, laboratory results, and death records, within a single secure environment.
- MACCS includes patient demographics, data on medicines prescribing and administration/supply, key biochemistry and haematology test results (e.g. kidney and liver function tests), data on hospital admissions and surgical procedures, and date and cause of death.
- The data resource provides longitudinal follow-up of the adult population (≥18 years of age) receiving medicines through NHS Scotland since 2010, covering ∼4.6 million individuals, and supports pharmacoepidemiological studies, drug-utilization research, pharmacovigilance projects, as well as health-services research.
- Approved researchers can apply through a streamlined process to access the linked MACCS data resource through established NHS Scotland governance processes, with data accessed within a Trusted Research Environment.

## Data resource basics

Healthcare in Scotland is delivered through a universal, publicly funded system operated by NHS Scotland, under which services are provided free at the point of care to all residents. This single-payer structure enables comprehensive population coverage and the routine collection of administrative health data at a national level [1–3]. NHS Scotland serves a population of ∼5.5 million people [1], encompassing both urban and rural settings and a wide range of socio-economic circumstances. Over recent years, electronic systems have been implemented to support the prescribing and dispensing/administration of medicines in both primary and secondary care [4, 5], to complement already-existing electronic systems providing a wide range of functionalities across the healthcare system [6, 7]. However, because medicines are prescribed in different healthcare settings, namely primary care, secondary care, and home care, information is captured in separate datasets reflecting the context of care delivery. Furthermore, medicine-related data are stored separately from other routinely collected healthcare data such as hospital or death records due to data being collected through various distinct electronic systems. While these electronic systems have strengthened clinical practice and patient safety within individual settings, the resulting fragmentation of data presents challenges for research, including attempts to understand patterns of medicine use and associated outcomes across the healthcare system as a whole [6–12]. The Medicines in Acute and Chronic Care in Scotland (MACCS) data resource was established in 2025 to address this gap by providing comprehensive, longitudinal data on medicine use across all healthcare settings in Scotland, pre-linked to a range of other healthcare datasets. While Hospital Electronic Prescribing and Medicines Administration (HEPMA) [5] captures hospital prescribing and medicines administration data and the Scottish Combined Medicines Dataset (SCoMeD) [7] integrates prescribing data across primary-care, secondary-care, and homecare settings, MACCS extends this framework by additionally linking medicine exposure to national datasets on hospital admissions, outpatient-clinic attendances, laboratory investigations, microbiology, intensive care admissions, disease registries (e.g. cancer and renal registries), and mortality records, thereby enabling longitudinal pharmacoepidemiological and outcomes research at the population scale. Record linkage is facilitated by the availability of a unique patient identifier in Scotland, allocated to every resident and included in all health and social-care records [13]. MACCS covers all adults (≥18 years of age) residing in Scotland who have received at least one prescription medicine since 2010; at the time of writing (February 2026), MACCS comprised data on ∼4.6 million individuals.

MACCS is based on existing routinely collected data and data use is governed through established NHS Scotland information-governance processes. Approval for the establishment of the MACCS data resource was obtained through the Public Benefit and Privacy Panel for Health and Social Care (PBPP-HSC) [14]; MACCS is held and maintained by Public Health Scotland (PHS) and is part of the PHS infrastructure, with initial development support provided by Health Data Research UK and the Scottish Government Pharmacy, Medicines and Therapeutics Division. MACCS is intended as a long-term, curated data resource contingent on the continued operation of national prescribing systems and public-sector funding arrangements, to be updated annually, for use by approved researchers within a Trusted Research Environment [15].

## Data collected

MACCS is derived entirely from routinely collected administrative healthcare data and integrates information from multiple existing national datasets within NHS Scotland. Individuals are included in MACCS on the basis of medicine exposure: the eligibility criteria for inclusion are being aged ≥18 years and having received at least one prescription medicine within NHS Scotland—dispensed through a community pharmacy since April 2009, administered in hospital since July 2022, and/or delivered directly to their home through the Medicines Home Care Services since January 2019. Extensive clinical, demographic, and laboratory information is available for included individuals through record linkage via the Community Health index (CHI) number—a unique person identifier that enables deterministic linkage across the datasets [13]. Record linkage is performed by the Electronic Data Research and Innovation Service (eDRIS) [16] within PHS and follows established information-governance procedures.

MACCS currently combines information from 14 different sources, thereby providing data on medicine use; medical conditions, including laboratory data; hospitalizations; deaths; and socio-demographics. National medicine datasets include the Prescribing Information System (PIS), holding information on medicines supplied in community pharmacies [4]; HEPMA, covering inpatient wards in hospitals (excluding systemic anticancer therapy and intensive care units) [5]; and Home Care Medicines (HCM), i.e. data on medicines prescribed in secondary care and delivered directly to a patient’s home (typically highly specialist medicines such as biologics) [17]. Disease registries such as the Cancer Registry (SMR06) [18] and the Scottish Renal Registry (SRR) [19] provide data on medical conditions; key biochemistry and haematology test results (e.g. kidney and liver function tests) are held in the Scottish Care Information (SCI) Store [20], while microbiology surveillance data stem from the Electronic Communication of Surveillance Scotland (ECOSS) [21]. Data on hospitalizations are provided through Scottish Morbidity Records (SMR) covering outpatient, inpatient, maternity, and mental healthcare (SMR00/01/02/04) [22–25]; and the Scottish Intensive Care Society Audit Group (SICSAG) [20] covering critical care. Death records from the National Records of Scotland (NRS) are available to provide data on mortality [26]. Patient demographics—including age, sex, health board, and urban–rural classification of residence, and the Scottish Index of Multiple Deprivation (SIMD) [27] are available across the datasets (Table 1). The SCI Store includes a wide range of biochemistry and haematology tests, but access to specific laboratory variables is typically defined at the project level based on research need and governance approvals. Work is in progress to add further data; in the first instance, Unscheduled Care Data comprising data from Emergency Departments, GP out-of-hours systems, and the Scottish Ambulance Service. Discussions are ongoing to also include primary-care data (data from GP records).

**Table 1:** Data provided in the MACCS data source.

Dataset	Dataset description	Type of data collected	Data coverage	Number of patients
**Medicine-use data**				
PIS [28]	Medicines prescribed and dispensed in primary care	Medicine name, formulation, strength; date prescribed, date dispensed; quantities prescribed and dispensed	April 2009–February 2025	5.4 million
HEPMA [5]	Medicines prescribed and administered in hospital	Medicine name, dose, route; date prescribed, date and time administered	July 2022–May 2025	800 000
HCM [29]	Medicines prescribed in secondary care and delivered directly to patients’ homes	Medicine name, quantity, supply date, indication	January 2019–August 2025	65 000
**Clinical information**				
SMR06 [18]	Details on cancers diagnosed in Scotland	Tumour type, morphology, date of diagnosis	April 2004–May 2024	775 000
SRR [19]	Details on patients with kidney disease	Kidney disease diagnoses, date/type of treatments, transplant records	April 2004–September 2025	6500
SCI Store [20]	Laboratory investigations—biochemistry and haematology	Test name, test date, test value	To be defined at the project level	To be defined at the project level
ECOSS [21]	Laboratory investigations—microbiology	Microbiological test results, specimen, organism, sensitivity, MIC	January 2019–December 2025	3.6 million
**Hospitalizations**				
SMR00 [22]	Outpatient-clinic attendances	Clinic speciality, referral reason, date of clinic attendance	April 2004–June 2025	4.9 million
SMR01 [23]	Hospital inpatient and day-case episodes	Admission date, admission reason (ICD-10), surgical procedure (OPCS-4), discharge date, length of inpatient stay	April 2004–June 2025	3.8 million
SMR02 [24]	Maternity care	Date of delivery, admission/discharge date, admission reason (in labour/after delivery at home), diabetes(Y/N)	April 2004–June 2025	594 000
SMR04 [25]	Mental health inpatient and day-case admissions	Admission/discharge date, reason for admission, admission type (emergency/routine)	April 2004–June 2025	149 000
SICSAG [13]	Intensive care unit (ICU) admissions	ICU admission date, organ support, interventions	January 2005–December 2024	419 000
**Mortality**				
NRS death records [26]	Civic registration system	Date of death, cause of death	April 2009–October 2025	938 000
**Patient demographics**				
CHI		Age, sex		
Various	Geographical information based on postcode	Health board of residence, urban–rural classification, deprivation (SIMD)		

In HCM, the supply date corresponds to the delivery date.

**Figure 1:**
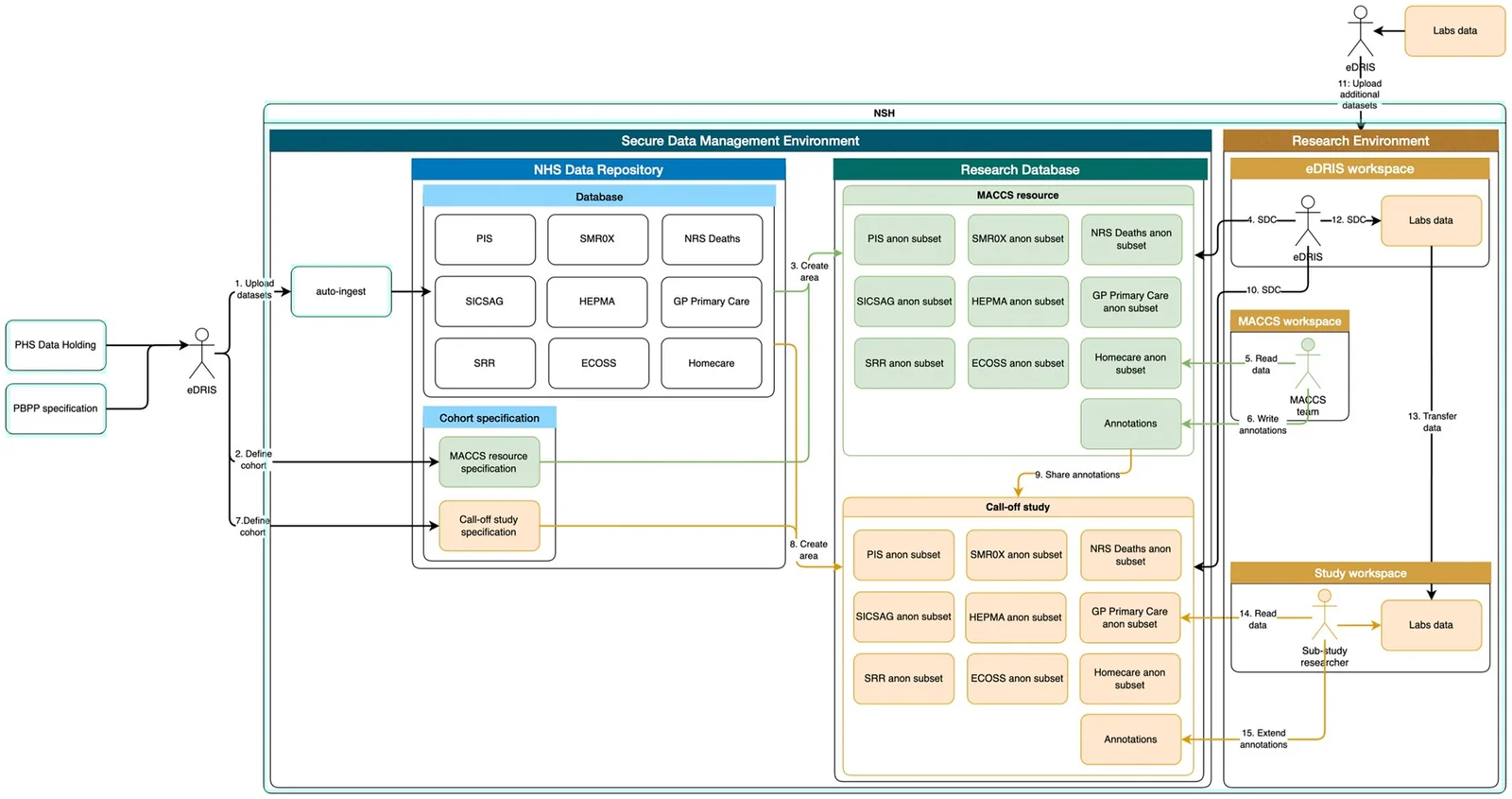
MACCS architecture schematic. This schematic illustrates the operation of the MACCS data resource. Source datasets are held within the National Safe Haven secure data environment and are managed by the eDRIS team through the NHS Data Repository Database, alongside the MACCS project specification code. To create the MACCS research environment, national datasets are linked, cleaned, and standardized in line with MACCS resource specifications. For each approved study, project-specific data views are generated from the core resource based on defined call-off requirements and made available to researchers through controlled analytical workspaces with integrated governance and auditing. Call-off contracts are individual contracts that fall under framework agreements. SMR0X, Scottish Morbidity Records for dataset 00/01/02/04/06; anon, annotation; SDC, Statistical Disclosure Control.

Source extracts from PHS datasets are ingested into a controlled staging layer in which original dataset structures are preserved and initial data-quality checks are performed (Fig. 1). Within the National Safe Haven—a secure environment used to enable researcher access to pseudonymized individual-level data—MACCS is organized via a structured database design (or schema) that defines how different types of information are stored, related, and queried. The core schema includes person-level tables that hold basic demographic information for each individual, alongside harmonized medicine-exposure tables derived from the PIS, HEPMA, and HCM, which record medicine prescribing and supply (where available) in a consistent format. Additional event-based tables capture linked information on hospital activity, laboratory results, disease registries, and mortality data. Data are updated at regular intervals through repeat data extractions from the source datasets; this approach preserves a consistent data structure while allowing new records to be incorporated over time. All records include the dates of relevant events, allowing researchers to follow individuals over time, subject to data availability, and to support longitudinal analyses, the use of look-back periods, and time-to-event study designs.

Variables are harmonized across datasets as far as possible (e.g. consistent variable naming and formatting); standardized coding systems are used where available and appropriate. These include e.g. British National Formulary coding [28] or medicines dispensed in community pharmacies and Dictionary of Medicines & Devices (dm+d) codes [29] for medicines prescribed and administered in hospital settings, both of which can be mapped to the Anatomical Therapeutical Chemical classification system [13]; and International Classification of Diseases 10th Revision (ICD-10) codes [30] to capture reasons for admission to hospital and causes of death. Dates are recorded by using standard ISO formats and clearly defined code lists are used for categorical variables.

## Data resource use

MACCS can support a wide range of projects in areas such as pharmacoepidemiology, pharmacovigilance, and health-services research due to the extent of data available. This may include the evaluation of prescribing practices across Scotland’s care settings, the tracking of real-world medicine use at a population level, and the assessment of patient adherence to medication, long-term safety monitoring of medication use, or assessing associations between medication use, laboratory markers, and clinical outcomes over time. Furthermore, MACCS and the infrastructure in which it sits can support various methodological approaches, including advanced statistical methods and the application of artificial intelligence tools.

An illustrative example of the current application of MACCS is a study examining high-dose antipsychotic prescribing in hospitalized patients. The aim of the study is to assess current prescribing practices and evaluate the association between high-dose antipsychotic prescribing and clinical outcomes among hospitalized patients; more specifically, objectives include calculating the prevalence of high-dose antipsychotic prescribing across different hospital settings; describing patient characteristics and clinical profiles of patients exposed to high-dose antipsychotic therapy; and analysing both short- and long-term effects of high-dose antipsychotic use (e.g. adverse events, subsequent hospitalizations, and mortality). MACCS facilitates the identification and characterization of prescribing patterns and patient exposure across care settings, in both primary and secondary care; moreover, MACCS provides access to crucial laboratory data as well as the required data on hospitalizations and death. Findings from this study will be used to inform the development of a subsequent project focused on designing an integrated computerized clinical-decision support tool to support safer antipsychotic prescribing in clinical practice (study protocol [31]).

MACCS is also currently being used to support a feasibility study evaluating real-world safety surveillance of targeted immune-modifying therapies across immune-mediated inflammatory disease in Scotland, commissioned by the Medicines and Healthcare products Regulatory Agency. By linking HCM data with hospital admissions, the Cancer Registry, and death records, MACCS enables the identification of new users of biologics and Janus kinase inhibitors, and the estimation of incidence rates for serious infections, major cardiovascular events, venous thromboembolisms, and malignancies. The work demonstrates the capacity of MACCS to underpin scalable, population-level pharmacovigilance within a national regulatory context.

Additional studies using MACCS are in various stages of planning. For instance, a study examining anticoagulant prescribing patterns over time and associated clinical outcomes has recently been approved; this study aims to develop and validate a prediction model to estimate the risk of severe bleeding in patients treated with direct oral anticoagulants in Scotland. The study will quantify how often serious bleeding events (hospitalization or death) occur and identify key demographic, clinical, and treatment-related risk factors. Using the MACCS resource, the study aims to create a robust multivariable model that estimates individual risk at treatment initiation, supporting clinicians to identify high-risk patients and guide safer, personalized prescribing and monitoring decisions. Another planned study will focus on the long-term effects of COVID-19 infections in the Scottish population, exploring medication use for psychiatric disorders following infection, describing treatment pathways, and assessing associated clinical outcomes.

## Strength and weaknesses

A major strength of MACCS is its population-scale coverage within a universal healthcare system, enabling the near-complete ascertainment of medicine prescribing, dispensing, administration, and/or supply for adults receiving NHS Scotland care. Because data are captured routinely whenever individuals interact with NHS services, which are provided free at the point of care to all residents, MACCS supports longitudinal analyses without the need for sampling and therefore minimizes selection bias commonly encountered in cohort studies. The integration of medicines data from different settings within a single, pre-linked resource allows the comprehensive assessment of medicine use and treatment pathways across NHS Scotland care. A further strength is the linkage of medicine-exposure data to a wide range of national clinical datasets, including hospital admissions, laboratory results, and mortality records. This enables the robust investigation of treatment outcomes, adverse events, and safety signals in real-world settings. The provision of data within MACCS through curated schema and harmonized variables reduces the technical burden associated with assembling multi-source datasets and supports reproducible research through consistent data structures and periodic refreshes. Finally, MACCS also benefits from a streamlined governance and access model. Approved researchers only have to apply once to access a pre-linked data resource within a secure Trusted Research Environment, rather than having to navigate multiple dataset-specific approvals. This improves efficiency while maintaining robust information governance, privacy protection, and disclosure control.

Limitations of MACCS mainly reflect those inherent in routinely collected administrative data. MACCS is not a whole-of-population resource, as inclusion depends on recorded medicine exposure within NHS Scotland (PIS, HEPMA, HCM) and individuals without recorded exposure are not included. The resource is also restricted to adults, limiting paediatric and transition-of-care research. Some variables may be incomplete or unavailable; e.g. the prescribing indication is not available in PIS or HEPMA and completeness of variables such as health boards and deprivation may vary across data settings. Although CHI-based deterministic linkage ensures high accuracy, emigration and deregistration information may vary, potentially resulting in minor overestimation of person-time calculations. Differences in data-collection purposes across systems introduce heterogeneity; for instance, HCM prescription dates may be approximated by using delivery dates; also the temporal coverage varies across datasets, fully integrated medicines data only being available from 2022 onwards may limit longitudinal analysis requiring complete cross-setting medicine exposure. Medicine data may also not reflect actual consumption and variation in recording practices may have introduced misclassification. In addition, some therapies, such as systemic anticancer treatments, are not fully captured due to separate specialist systems. Finally, routine quality checks prior to making data available to researchers introduce a time lag, with potential implication for time-sensitive research. Despite these limitations, MACCS remains a highly comprehensive resource that is currently available for the population-scale evaluation of medicine use and associated outcomes in Scotland.

## Data resource access

MACCS is not an open-access dataset due to the inclusion of person-level health data. Consequently, no public web-based download is available. Access to the data is granted only for approved research projects and is provided exclusively within secure Trusted Research Environments in accordance with NHS Scotland information-governance requirements. Researchers seeking access to MACCS for research purposes must submit an application through eDRIS at PHS; all requests are subject to review and approval by PBPP-HSC [14].

Supporting documentation for MACCS, including variable lists and data dictionaries describing variable definitions, derivation rules, and coding systems, is available to approved users. These materials are provided as part of the data-access process and are maintained by PHS. Variables are presented in a structured, analysis-ready format, with consistent naming conventions applied across the data sources; variable names typically follow clear, descriptive rules, using standardized prefixes and suffixes to distinguish patient-level characteristics, medicine-exposure variables, and derived measures. No specialist software is required to access MACCS beyond what is provided within the Trusted Research Environment. Approved users can analyse the data by using standard analytical tools such as R and Python; analyses can be conducted remotely, although researchers need to be UK-based to be able to access the remote server. Direct access to MACCS data within the Trusted Research Environment is currently restricted to UK-based researchers due to data-governance and secure-infrastructure requirements. However, international researchers can participate through formal collaboration with UK-based institutions or NHS partners, who can host analyses within the secure environment. Such collaborations support wider national and international research while ensuring compliance with data-protection and confidentiality requirements.

Enquiries regarding the MACCS data resource, including questions about data content, access processes, and potential collaboration, can be directed to the corresponding author: Cosmika Goswami, University of Strathclyde, at cosmika.goswami@strath.ac.uk or to PHS directly at phs.eDRIS@phs.scot.

## Ethics approval

The development and setting-up of MACCS was approved through the Public Benefit and Privacy Panel for Health and Social Care. Additional ethical approval was not required. No data were collected or generated specifically for this project. PBPP-HSC approval typically takes a month or two, depending on the study complexity, and data provision following approval generally takes a few weeks to a month, depending on the dataset complexity and linkage requirements.
